# Learning how to perform ultrasound-guided interventions with and without augmented reality visualization: a randomized study

**DOI:** 10.1007/s00330-022-09220-5

**Published:** 2022-11-09

**Authors:** Nadja A. Farshad-Amacker, Rahel A. Kubik-Huch, Christoph Kolling, Cornelia Leo, Jörg Goldhahn

**Affiliations:** 1 Radiology, Balgrist University Hospital, University of Zurich, Forchstrasse 340, 8008 Zurich, Switzerland; 2 Institute of Radiology, Department of Medical Services, Kantonsspital Baden, Baden, Switzerland; 3 Institute of Translational Medicine, Department of Health Sciences and Technology, Eidgenössische Technische Hochschule (ETH), Zurich, Switzerland; 4 Department of Gynaecology and Obstetrics, Kantonsspital Baden, Baden, Switzerland

**Keywords:** Augmented reality, Ultrasound, Learning, Biopsies, Puncture

## Abstract

**Objectives:**

Augmented reality (AR), which entails overlay of in situ images onto the anatomy, may be a promising technique for assisting image-guided interventions. The purpose of this study was to investigate and compare the learning experience and performance of untrained operators in puncture of soft tissue lesions, when using AR ultrasound (AR US) compared with standard US (sUS).

**Methods:**

Forty-four medical students (28 women, 16 men) who had completed a basic US course, but had no experience with AR US, were asked to perform US-guided biopsies with both sUS and AR US, with a randomized selection of the initial modality. The experimental setup aimed to simulate biopsies of superficial soft tissue lesions, such as for example breast masses in clinical practice, by use of a turkey breast containing olives. Time to puncture(s) and success (yes/no) of the biopsies was documented. All participants completed questionnaires about their coordinative skills and their experience during the training.

**Results:**

Despite having no experience with the AR technique, time to puncture did not differ significantly between AR US and sUS (median [range]: 17.0 s [6–60] and 14.5 s [5–41], *p* = 0.16), nor were there any gender-related differences (*p* = 0.22 and *p* = 0.50). AR US was considered by 79.5% of the operators to be the more enjoyable means of learning and performing US-guided biopsies. Further, a more favorable learning curve was achieved using AR US.

**Conclusions:**

Students considered AR US to be the preferable and more enjoyable modality for learning how to obtain soft tissue biopsies; however, they did not perform the biopsies faster than when using sUS.

**Key Points:**

• *Performance of standard and augmented reality US-guided biopsies was comparable*

• *A more favorable learning curve was achieved using augmented reality US.*

• *Augmented reality US was the preferred technique and was considered more enjoyable*

## Introduction

Within the last few years, virtual reality and augmented reality (AR) have been increasingly used in the fields of entertainment and gaming. More recently, they have also been implemented in the field of medicine, particularly for training [1–4] but also clinically, such as in the fields of surgery and medical imaging [5–15]. Various head-mounted displays (HMDs), such as Google Glass and Microsoft HoloLens, are commercially available. These powerful tools generate a high degree of flexibility, enabling the operator to visualize virtual image content anywhere within the room, including superimposing it over any object or individual in that room. Thus, HMDs can be used both for educational purpose and clinically.

For example, superimposing a computed tomography (CT) image over anatomical structures enables the operator to visualize deeper structures that would otherwise not be visible on just the surface. This, in combination with planned navigation, has been used to guide injections or biopsies [6–9, 16–19], assist hepatic cancer surgery [20], navigate pedicle screw positioning during surgery [14, 21, 22], and assist extremity reconstruction surgery [11, 15, 23]. Furthermore, AR and VR have increasingly been used for educational purposes. AR techniques can provide a virtual learning world (virtual reality) in medical education and training, enabling simulation of clinical skills and thus facilitating preparation of medical professionals for the real world. Examples include practicing interventions or surgeries in a virtual environment. AR techniques can also be used to enhance the reality of practical skills (mixed reality). This can enable students to learn how to perform procedures more quickly and improve more rapidly thereafter. AR techniques have been implemented for some specific tasks, such as providing navigated guidance for pedicle screw positioning [5]. There are many other applications of AR in medical education [2, 24].

In this study, we assessed AR US, which has the following capabilities: Images seen through a HMD can by using a voice command either be set steady, to follow the eyes, or be positioned at a certain location, for example below an ultrasound (US) probe. Furthermore, use of tracking objectives by implementing a quick response (QR) code at the US probe can enable the image to follow the tracked US probe. The US image can thus be displayed directly below the US probe in the exact real time anatomical position (in situ image). In a previous phantom study, the performance of inexperienced operators was found to be superior when they used an AR in situ technique [6]. Therefore, we hypothesized that students with only minor or moderate US experience would also perform better with an AR US technique than with standard US (sUS). Investigating this hypothesis was the primary purpose of this study. Furthermore, we aimed to study the student’s learning experience with both modalities.

## Materials and methods

### Operators

The institutional review board approved this prospective, randomized study, and the participants gave their consent to participation.

The study cohort comprised 44 medical students (28 women, 16 men) who had no experience in performing biopsies but had completed a standard US course (16 lessons).

### Soft tissue/breast phantom (as used by the Minimally Invasive Breast Biopsies Working Group of the Swiss Society of Radiology)

**Fig. 1:**
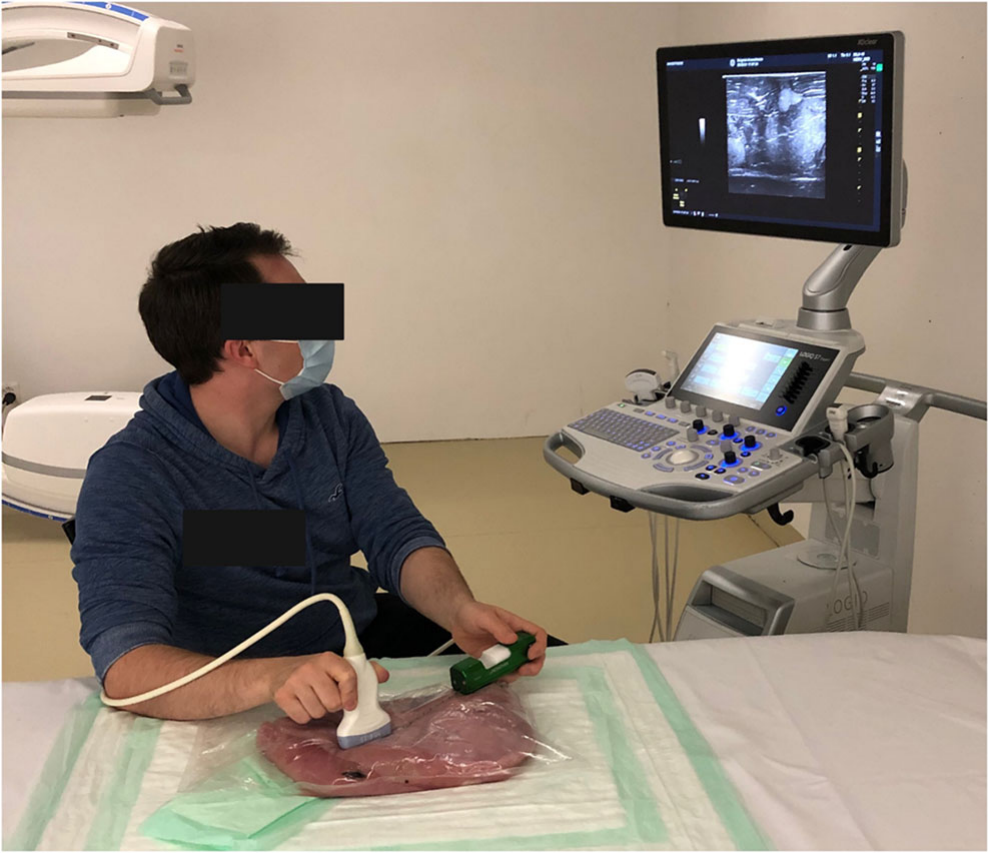
Ultrasound guided biopsy using standard US (sUS). Note the turkey breast containing olives simulating a breast with soft tissue lesions

The breast phantoms comprised four turkey breasts (each 2–3 kg) filled with olives (*n* = 15) to simulate breast tissue lesions. The phantoms were vacuum packed in transparent, thick plastic wrap (Fig. 1).

### US techniques

The sUS system used was a GE Logiq S7 expert (General Electric) with a matrix linear probe (ML 6-15) (Fig. 1).

**Fig. 2:**
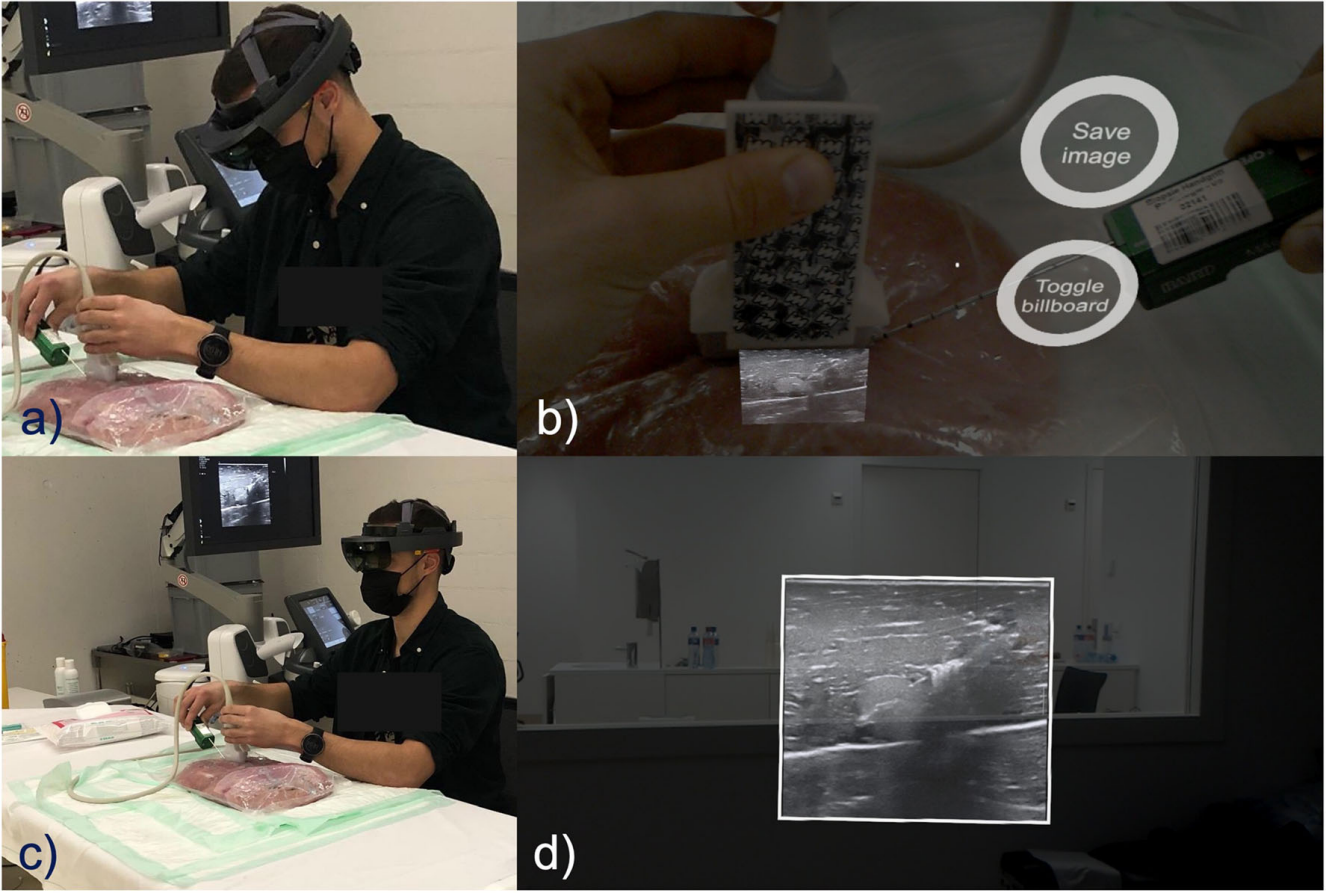
Ultrasound guided biopsy using augmented reality (AR US). **a**, **b** Using a QR code attached to a US transducer to track a US probe within the room, an in situ actual-sized image is projected directly below the US probe at the exact anatomical position in size real time. **c**, **d** Lifting the head initiates display of a second bigger image (same size as on an ultrasound screen) that follows the eyes of the operator

The AR in situ US was composed of a conventional US system (SuperSonic Aixplorer Ultimate) with a linear probe (SL 18-5) and custom-developed software that transmitted the US image to an industry-grade head-mounted AR display (Microsoft Hololens). Furthermore, a QR code that tracked the probe to any position within the room was attached to the US probe (Fig. 2a, b). Two images were visible when using the head-mounted AR display. One image was positioned by the QR code tracking system at the exact anatomical location that was to be examined in real time, this being below the ultrasound probe (in situ image) (Fig. 2b).

The location of the image to be displayed below the US probe at the exact anatomical position was calculated using the world coordinate system of the head-mounted device and adjusted for a relative offset generated by the US probe head, marked with the QR code. The geometrical properties of the US probe head are exactly known and, together with the applied QR tracking code, an initial calibration to anchor the in situ image correctly in space, as already reported in our previously performed phantom study [6].

This in situ image was relatively small, showing the actual dimensions without magnification (1:1); however, it supports the direct eye-hand coordination for the interventional steps by avoiding size and spatial abstraction (Fig. 2b). When the operators lifted their heads, they could see a magnified version of the same image, similarly to that shown on the sUS screen, but differing in that it followed the operator’s eye motion (Fig. 2d).

### Briefing of the operators before the procedure performance

Before the experiments, students were instructed during 15 min in a standardized fashion regarding the usage of the probe, locating and targeting the lesion, as well as performing the biopsy.

### Study workflow

Each participant was required to puncture the turkey breast containing the olive-simulated lesions with a 14G disposable core biopsy needle (Bard Magnum) used in concert with a Bard Magnum Biopsy instrument. The students were asked to perform the procedure three times using AR in situ US and three times using sUS. The initial modality was selected randomly, but balanced, 22 participants starting with AR in situ US and 22 with sUS.

AR in situ US and sUS were performed in two different rooms with two different supervisors. For sUS, the supervisor was a radiologist who was specialized in imaging of women, whereas for AR US the supervisor was a gynecologist who was specialized in female breast biopsies. Both supervisors had more than 20 years of experience with US-guided biopsies. US settings (including focal zone) were prepared by supervisors, before start of the operator’s task.

Primary endpoints were the speed and the accuracy with which the target was punctured. The times taken to identify a target(s) and to puncture that target(s) were measured. In addition, whether the biopsy was successful (more than > 25% of the core biopsy needle filled with olive pulp) or unsuccessful (no or < 25% olive pulp in the core biopsy) was documented.

Secondary endpoints were educational experience, relationship to manual coordination in other activities, and ergonomics efficiency. To enable assessment of the secondary endpoints, all participants filled out a questionnaire concerning their coordinative skills, before the interventions. This questionnaire included items such as history of sport activities with hand-eye coordination (such as tennis), playing an instrument, video gaming, and making a self-assessment of their coordinative skills (ranging from 0 to 5). Having a gaming history was defined as regularly playing video games at least once a month.

The students also filled out a second questionnaire after the interventions to report which modality they preferred (AR US vs. sUS), which provided a more enjoyable learning experience, and which image they preferred when using the AR US, and which modality was more ergonomically efficient.

Study data were collected and managed using REDCap electronic data capture tools hosted at Balgrist University Hospital [25].

### Statistical analysis

Statistical analyses were performed using IBM SPSS Statistics for Windows, Version 27. Descriptive statistics were used to express career aspiration by gender, self-assessment of manual skills, history of playing video games, significant coordinative skills, and number of missed hits using sUS and AR US. Further descriptive statistics were used to assess which of the two techniques was preferred, easier to learn with, and fun to learn with.

Medians and ranges were used to report non-parametric data, such as time taken to puncture the targets. Wilcoxon matched-pairs signed rank tests were applied to test for any significant difference between the two US techniques. A *p*-value < 0.05 was considered to denote statistical significance.

## Results

The AR US technique was considered more enjoyable to learn with by 79.5% of the participants and 61% indicating that they found the AR technique to be more ergonomically efficient. The AR US technique was preferred by 59% of the participants. In contrast, only 36% preferred the sUS technique and 5% were undecided.

**Fig. 3:**
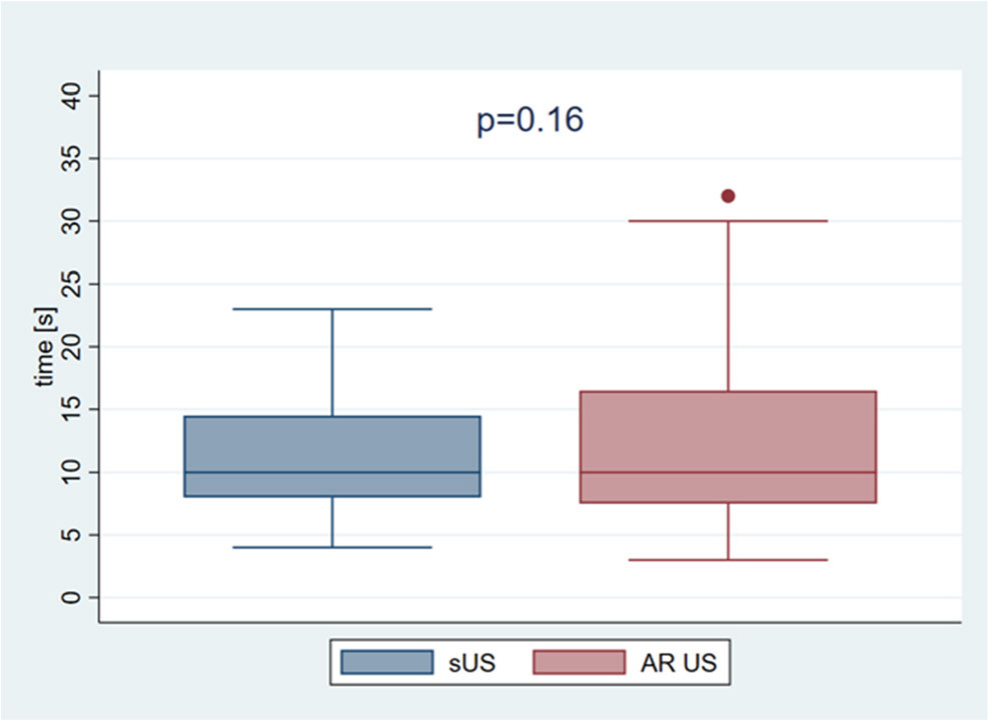
Median times(s) taken to puncture the target using standard US (sUS) and augmented reality (AR) US did not differ significantly

The overall times taken to identify and puncture a target with AR US and sUS did not differ significantly (median [range] to identify: 27.5 s [5–297 s] versus 31.5 s [4–178 s], *p* = 0.57 and median [range] to puncture: 17.0 s [6–60] and 14.5 s [5–41], *p* = 0.16, Table 1, Fig. 3).

**Table 1:** Times taken to identify and puncture a target with AR US and sUS

Time taken to puncture [s]	sUS			AR US			*p* value
	Median	Min	Max	Median	Min	Max	
First attempt	12.5	3	49	17.0	5	111	0.05
Second attempt	15.0	3	68	12.5	3	95	0.23
Third attempt	10.0	2	57	11.0	3	60	0.04
Median of the three attempts	14.5	5	41	17.0	6	60	0.16
Median of last two attempts*	12.0	4.5	45.5	13.5	4.0	51.0	0.35
Mean of all three attempts	13.8	4.7	46.7	15.0	6.0	49.0	0.05

*Treating the first attempt as a test run

*sUS* standard ultrasound, *AR US* augmented reality ultrasound, *max* maximum, *min* minimum, *s* seconds

The mean times taken to puncture the target also did not differ significantly between using AR US or sUS (median [range]: 15.0 s [6–49 s] versus 13.8 s [5–47 s], respectively; *p* = 0.05; Table 1). When we treated the first attempt at each US technique as a test and excluded those values from the statistical analysis, there was an even smaller difference between AR US and sUS (median [range]: 13.5 s [4–51 s] and 12.0 s [5–46 s], respectively; *p* = 0.35; Table 1).

**Fig. 4:**
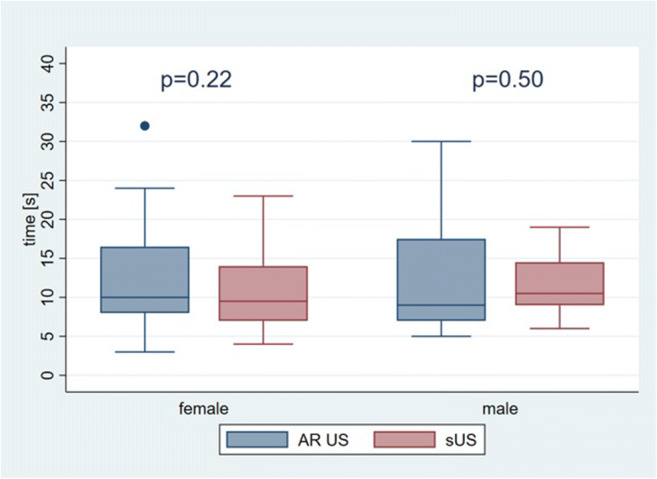
There were no gender-specific differences between using standard US (sUS) or augmented reality (AR) US in puncturing the target

There were also no significant differences in achievement when the two techniques were compared by gender (*p* = 0.22 vs. *p* = 0.50; Fig. 4). Unsuccessful punctures were rare (*n* = 11 in both techniques) and their frequency did not differ significantly between the two US techniques (*p* = 0.71).

Furthermore, overall, a better learning curve from the first to the third attempt was achieved when using the AR technique than when using sUS (Table 1).

**Fig. 5:**
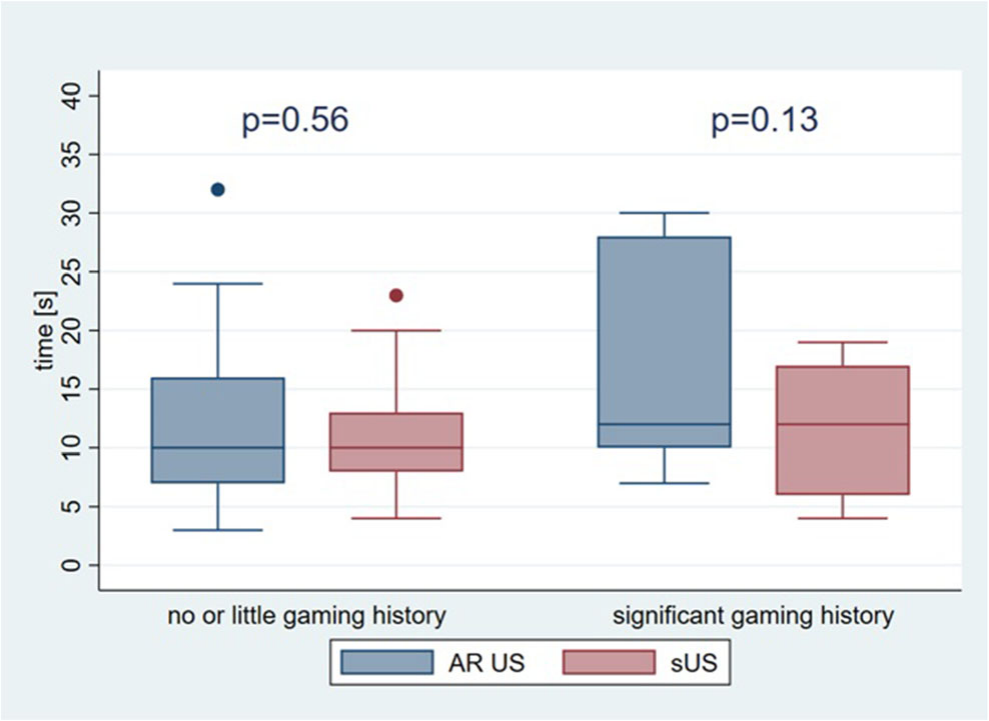
Comparison of the median time to puncture the targets with augmented reality ultrasound (AR US) and standard US (sUS), stratified by personal history of gaming

We considered that 86% of our participants had significant coordinative skills based on having played a musical instrument, having played sports for years, or having a gaming history. Only 16% of our participants had a gaming history. Individuals with a significant history of gaming did not perform significantly better when using the AR technique than when using the sUS technique (16.3 s versus 11.9 s; *p* = 0.13) (Fig. 5).

**Fig. 6:**
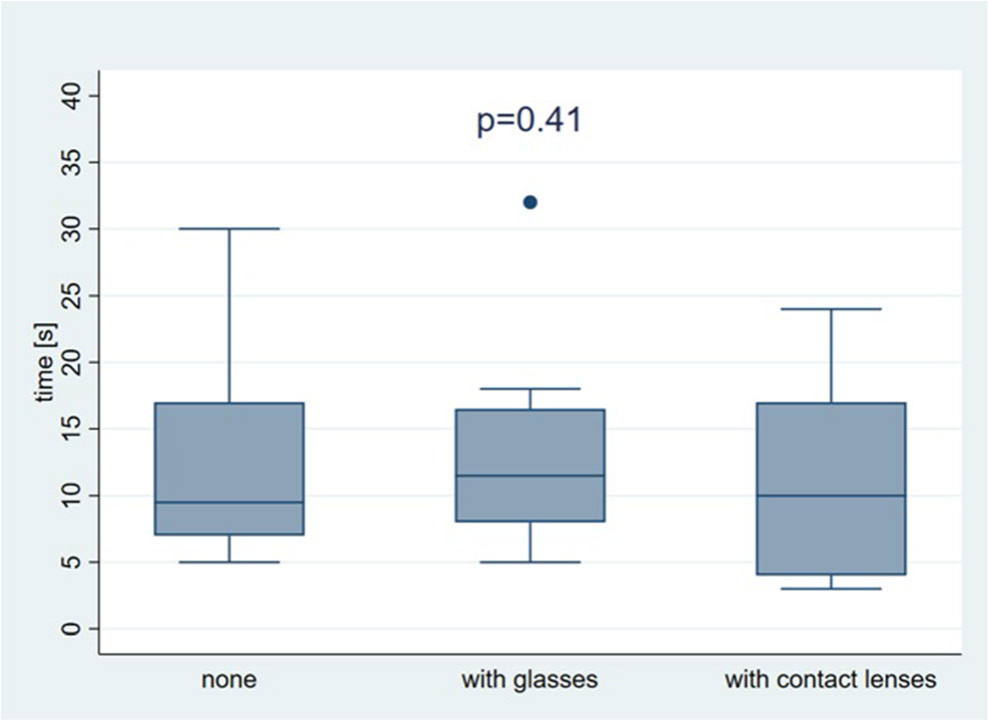
Comparison of the median time to puncture the targets with augmented reality ultrasound (AR US), stratified by need for visual aids; the differences are not statistically significant (*p* = 0.41)

A need for visual aids such as lenses or glasses had no detectable influence on outcomes when using the AR technique (Fig. 6).

## Discussion

Use of AR technology is increasing in several fields, including medicine. Early adaptations include image-guided infiltrations [6–10, 16, 26], surgery [5, 11–15], and other procedures benefitting from imaging guidance. Our aim was to determine whether AR US would be of value in training individuals who had not previously been taught how to perform US-guided biopsies and had not performed any such procedures. We found that, despite having no experience with AR techniques, operators were as successful at performing AR US-guided biopsies as they were at performing sUS-guided biopsies. However, participants achieved a better learning curve with the former and reported that they preferred it and found it more enjoyable.

We failed to confirm that our initial hypothesis, which was that inexperienced operators would perform biopsies of soft tissue lesions faster when using AR US than when using sUS, as demonstrated in a previously reported study [6]. Possible explanations for our failure to demonstrate that AR US was superior to sUS when puncturing lesions include the following: First, the participants in the present study were familiar with sUS, having completed a basic sUS course and also the 15-min introduction given by the supervisor was only on sUS. In contrast, the surgeons in the previous study had absolutely no experience of either sUS or AR US [6]. Moreover, no participants in the present study had previously seen or used an AR US technique and most of them had not previously worn HMDs, while the surgeons in our former study were used to wear HMDs.

Second, in this study, the HMD application displayed two US images, one below the US probe at the exact anatomical location (in situ image), and a second image being the same but magnified and visible only when the head was lifted. The in situ image appeared rather small, as its actual dimensions were being viewed. The magnified US image, which was displayed when the head was lifted, was approximately the same size as on a sUS monitor and this magnified image followed the operator’s eyes. Many of the participants were probably initially overwhelmed by the two images, as indicated by the fact that they tended to look up and down repeatedly, presumably deciding which image they preferred for performing the biopsy. With the benefit of hindsight, we should have trained the participants in the AR US technique for better comparability. We tried to compensate for this issue by performing stratified analysis.

Third, in the study cited above [6], the operators were not taught to hold the US probe correctly, whereas in the present study the supervisors instructed the students in correct handling of the ultrasound probe. If they had not, the AR in situ technique would have appeared to be superior, because turning the US probe (switching right to left) automatically turns the ultrasound image to the correct anatomical location, whereas it is displayed incorrectly on the US monitor (turned) when using sUS.

Other limitations of the present study were that the supervisors did not change the modality and may have influenced the operators. Additionally, the turkey breast phantoms were not identical in that the olives were inserted randomly. Thus, the ease of obtaining a biopsy may have varied between the prepared phantoms. Further, some air artifacts might have influenced the operators as for 44 operators only four turkey breast were prepared. However, as more than 15 olives were inserted in each turkey breast we think, that the artifacts were of minor relevance. However, given that we used four different breast phantoms, the differences were hopefully distributed approximately equally. Of note, not all four breast phantoms were punctured with both modalities: they were punctured by all operators, but each with only one modality. Retrospectively, it would have been preferable to exchange both the supervisors and breast phantoms between the two modalities. Also, the fact that we had two different US apparatus could have influenced our results. However, we believe that this is negligible, considering that the lesions were easily visible with both US apparatus, and similar US probes were used.

Even with all these limitations having deleterious effects on the AR US data, the participants did not generally perform the US-guided biopsies significantly more slowly and the learning curve was better for AR US than for sUS.

Detailed analysis of gender distribution showed a tendency for women to perform the biopsies more slowly than their male colleagues when using the AR US technique; however, this difference was not statistically significant. This trend may have been related to the fact that male operators were more comfortable with the AR US technique and some of them had previously worn HMDs, probably associated with gaming histories. Although former studies have found a possible association between experience with gaming and superior performance of laparoscopic surgery [27], we did not identify any significant differences between gamers and non-gamers in performing either technique.

Interestingly, although puncturing with AR US was performed more slowly, most participants preferred the AR US technique and reported that it provided a better learning experience. The latter observation is concordant with several reports describing the advantages of 3D teaching and learning by AR use and is of utmost importance [2, 20, 24, 28, 29]. Providing interesting and enjoyable learning experiences is very important for improving learning capacity. Studies in other specialties have shown that different types of simulation can significantly shorten training or steepen the learning curve, particularly because of the specific 3-D cognition that is needed when performing US-guided interventions [30, 31]. Learning methods, such as AR, that provide interesting and enjoyable learning experiences are important tools for improving learning capacity. However, AR US might be promising, not only for educational purposes, but also for clinical purposes. Besides ergonomic aspects, AR US might increase safety, efficiency, and efficacy. For example, a wireless US probe together with a head-mounted device and voice commands potentially increases mobility and sterility. Further, inclusion of a virtual needly guide might increase precision accuracy. Cadaveric and clinical studies are initiated at our institution using such more advanced techniques.

The present data indicate that operators who have completed a basic sUS course perform US-guided biopsies similarly whether using AR US or sUS. However, most students preferred the AR US technique and the learning curve was steeper when using AR US. Further development of AR-based techniques may shift the equilibrium towards broader use of AR US. This evolution should be accompanied by innovation and research.

## Abbreviations

AR US: Augmented reality
CT: Computed tomography
HMD: Head-mounted display
sUS: Standard US

## Declarations

### Guarantor

The scientific guarantor of this publication is Nadja A. Farshad-Amacker.

### Statistics and biometry

Two of the authors have significant statistical expertise (ChK and JG).

### Methodology

• prospective

• experimental study

• multicenter study
